# Bear Skin

**Published:** 1891-12

**Authors:** R. A. Chudleigh


### BEAR SKIN.

The first question is what to do with it. A mat or hearth rug is at once suggested as one of the most common uses to which a skin can be put. Yet there are many objections to placing it in any low or horizontal position; the fur gets turned the wrong way, dust and dirt rapidly accumulate, the margins get rolled up, and an incurable look of untidiness comes over the whole thing. A vertical position, with the head upwards so that the trend of the hair is downwards, is much more to my own taste. It may either occupy some blank panel or piece of dry wall, or it may be utilized on the back of one of the old fashioned high backed chairs, the vigor of whose rough carving may be much mitigated thereby. Unless a skin is thoroughly cured, it is not well to use it as clothing; the imperceptible evaporation and warmth of the wearer will certainly bring out objectionable odors with great power. A skin rug thrown loose over the back of a chair has been my ordinary seat for full ten years, and I have no fault to allege against it. Next, as to curing. I have sought in vain for an amateur method of tanning a fur so that it may be pliant, soft, sweet, and noiseless. My own productions always have some crackle and stiffness in them. Nevertheless, I know what I shall do if ever I have again to face the querist's problem of making a badly cured skin inoffensive. There is a preparation of tannin mixed with various antiseptics, and pleasantly scented, which chemists sell for application to sore throats and relaxed tonsils. I shall fix the skin on a board and apply this astringent fluid a little at a time to small portions of the skin. I am rather ashamed of recommending a plan which I have not proved; but if one proceeds gently it is hard to see how any harm can be done, and I expect good somewhat confidently.—R. A. CHUDLEIGH, *Wimborne.*

